# Extensive chondroid bone in juvenile duck limbs hints at accelerated growth mechanism in avian skeletogenesis

**DOI:** 10.1111/joa.13109

**Published:** 2019-10-31

**Authors:** Edina Prondvai, P. Eckhard Witten, Anick Abourachid, Ann Huysseune, Dominique Adriaens

**Affiliations:** ^1^ Department of Biology, Evolutionary Morphology of Vertebrates Ghent University Ghent Belgium; ^2^ MTA-MTM-ELTE Research Group for Paleontology Budapest Hungary; ^3^ Department of Biology, Evolutionary Developmental Biology Ghent University Ghent Belgium; ^4^ Département Adaptations du Vivant UMR 7179 Muséum National d'Histoire Naturelle – CNRS Paris France

**Keywords:** accelerated skeletogenesis, avian precocial–altricial development, cartilage‐bone intermediate, duck diaphyseal osteohistology, posthatching periosteal growth

## Abstract

Modern altricial birds are the fastest growing vertebrates, whereas various degrees of precocity (functional maturity) result in slower growth. Diaphyseal osteohistology, the best proxy for inferring relative growth rates in fossils, suggests that in the earliest birds, posthatching growth rates were more variable than in modern representatives, with some showing considerably slow growth that was attributed to their assumed precocial flight abilities. For finding clues how precocial or altricial skeletogenesis and related growth acceleration could be traced in avian evolution, as a case study we investigated the growing limb diaphyseal histology in an ontogenetic series of ducks which, among several other avian taxa, show a combination of altricial wing and precocial leg development. Here we report the unexpected discovery that chondroid bone, a skeletal tissue family intermediate between cartilage and bone, extensively contributes to the development of limb bone shaft in ducks up to at least 30 days posthatching age. To our knowledge, chondroid bone has never been reported in such quantities and with an ontogenetically extended deposition period in post‐embryonic, non‐pathological periosteal bone formation of any tetrapod limb. It shows transitional cellular/lacunar morphologies and matrix staining properties between cartilage and woven bone and takes a significant part in the diametric growth of the limb bone shaft. Its amount and distribution through duckling ontogeny seems to be associated with the disparate functional and growth trajectories of the altricial wings vs. precocial legs characteristic of duck limb development. The presence of isogenous cell groups in the periosteal chondroid bone implies that cartilage‐like interstitial growth took place before matrix mineralization complementing appositional bone growth. Based on these characteristics and on its fast formation rate in all previously reported normal as well as pathological cases, we suggest that chondroid bone in ducks significantly accelerates diametric limb bone growth. Related to this growth acceleration, we hypothesize that chondroid bone may be generally present in the growing limb bones of modern birds and hence may have key skeletogenic importance in achieving extreme avian growth rates and placing birds among the fastest growing vertebrates. Thus, we encourage future studies to test this hypothesis by investigating the occurrence of chondroid bone in a variety of precocial and altricial bird species, and to explore the presence of similar tissues in the growing limbs of other extant and extinct tetrapods in order to understand the evolutionary significance of chondroid bone in accelerated appendicular skeletogenesis.

## Graphical abstract

In our study we report the unexpected discovery that chondroid bone, a cartilage‐bone intermediate tissue, extensively contributes to diametric limb bone growth in ducks up to late ontogeny (≥30 days posthatching age). Its protracted deposition period, spatial and temporal distribution, and cartilage‐like properties all suggest that chondroid bone plays a key role in accelerating avian skeletogenesis and thereby placing birds among the fastest growing extant vertebrates.

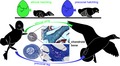

## Introduction

**Figure 1:**
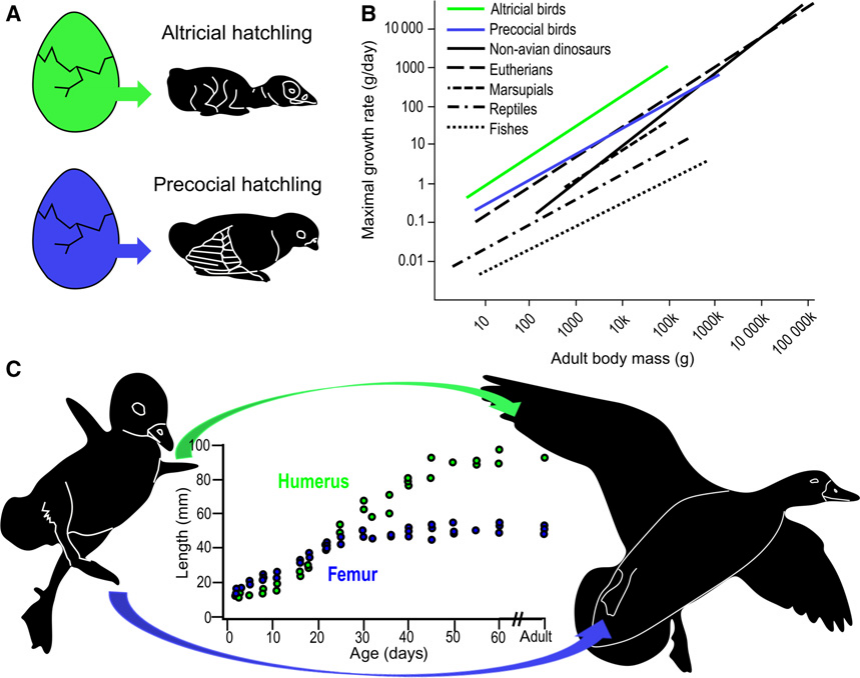
Precocial and altricial development in birds and related growth rate differences. (A) Schematic drawing contrasting the helpless, embryo‐like altricial and the functionally mature precocial hatchlings. (B) Growth rates in altricial (green) and precocial (blue) birds in relation to other vertebrates (modified after Erickson et al., 2001). (C) Ducklings with combined altricial wing and precocial leg development and the related differences in humeral (green) and femoral (blue) growth trajectories (growth diagram modified after Dial & Carrier, 2012).

The fastest growing living vertebrates are found among modern birds (Case, 1978) (Fig. 1). Posthatching growth rates are highest in altricial birds which have helpless, almost embryo‐like hatchlings, whereas various degrees of hatchling precocity, i.e. functional maturity, result in slower growth (Starck, 1993, 1994; Starck & Ricklefs, 1998; Starck & Sutter, 2000; Erickson et al. 2001) (Fig. 1A,1). High growth rates up to the altricial extreme, however, have not universally characterized birds throughout their evolution. Diaphyseal osteohistology, the best proxy for inferring relative growth rates in fossils (de Ricqlès et al. 2004; Padian & Lamm, 2013), suggests that growth decelerated in some early birds, such as *Archaeopteryx* or enantiornithines, compared with their non‐avian theropod ancestors. Although neither the ontogenetic onset of growth deceleration nor the relative duration of slow growth along their developmental trajectory is clear, it has been suggested that this slow growth is related to their superprecocity, i.e. the capacity to fly soon after hatching (Chinsamy & Elzanowski, 2001; Erickson et al. 2009; Xing et al. 2017). On the other hand, fossil ornithuromorphs, a more derived avian clade including modern birds, seem to have either retained or regained faster growth already in the Cretaceous (Chinsamy‐Turan et al. 1998; Wilson & Chin, 2014; O’Connor et al. 2015). How skeletogenesis enables growth acceleration events through avian evolution to the extreme seen in modern birds remains elusive due to insufficient data on the diaphyseal osteohistology of extant growing birds in the context of the precocial–altricial developmental spectrum. The degree and pattern of ossification and the relative extent and histology of epiphyseal cartilage are identified skeletal correlates of precocial–altricial limb bone development and associated growth rates (Starck, 1994; Horner et al. 2000, 2001; Chinsamy & Elzanowski, 2001; Montes et al. 2005). However, unmineralized tissues such as growing epiphyseal cartilage, are barely preserved in fossils, hampering such assessments in extinct taxa. By contrast, diaphyseal bone cortex is the most widely preserved, studied, and compared region in fossil long bones and also reveal the most complete primary growth record (Padian & Lamm, 2013). Thus, long bone diaphysis in modern birds is a valuable, albeit largely unexploited, source of information to explore how precocial–altricial skeletogenesis and related growth acceleration could be characterized and then traced in avian evolution.

As a first‐step case study, we investigated diaphyseal osteohistology in the limbs of growing Rouen ducks (*Anas platyrhynchos domesticus*), a special domestic breed that retains many wild‐type (mallard‐like) characters. Ducks, just like shorebirds, are characterized by a combined altricial wing−precocial leg development, i.e. by disparate growth and functional trajectories of the fore‐ and hindlimb elements (Castanet et al. 1996; Dial & Carrier, 2012) (Fig. 1C). The differential rates of wing and leg skeletogenesis in ducks are thus void of confounding interspecimen differences, and provide an excellent opportunity for identifying diaphyseal osteohistological traits associated with the respective growth burst period of the limb bones.

We used five specimens of Rouen ducks that were raised and euthanized for a previous study at 4, 8, 30, and 50 days posthatching age (dph) (Khabazi, 2017), representing early, mid‐way, and close to fledging ontogenetic stages. We sampled the humerus, radius, and ulna of the wing bones, and the femur, tibiotarsus, and tarsometatarsus of the leg bones for preparing transverse histological sections of the mid‐diaphyses. In this study, we primarily used demineralized, paraffin‐embedded sections with Toluidine blue (TB), Masson’s trichrome (MT), Alcian blue/haematoxylin (ABH), and Picro‐Sirius red (PSR) staining to maximize the efficiency of visual tissue identification. Undemineralized petrographic sections were also prepared for comparing corresponding tissue appearance acquired with the two techniques, those aiding future comparisons with petrographic sections of fossils and those for measuring growth‐related microanatomical parameters (Supporting Information Fig. S1).

Polarized light microscopic study of these sections has led to the unexpected discovery that chondroid bone, a skeletal tissue family intermediate between cartilage and bone (Beresford, 1981; Hall & Witten, 2007; Witten et al. 2010; Hall, 2014), extensively contributes to the diametric limb bone growth up to ≥30 dph. To our knowledge, chondroid bone has never been reported in such quantities and with such an extended post‐embryonic deposition period in non‐pathological periosteal bone formation of any tetrapod limb. This tissue shows transitional cellular/lacunar morphologies and matrix staining properties between cartilage and woven bone, and its distribution through ontogeny roughly parallels the disparate altricial and precocial growth trajectories characteristic of duck limbs (Dial & Carrier, 2012). These characteristics and its fast formation in all reported cases (Hall, 2014) suggest that chondroid bone accelerates limb growth burst in ducks and possibly in other birds, and may be a key element in ensuring the extremely rapid appendicular skeletogenesis, and hence in placing birds among the fastest growing vertebrates.

## Materials and methods

### Experimental design – acquisition context of duck specimens

For a previous muscle developmental study (Khabazi, 2017), 99 Rouen ducks purchased from the commercial market were raised from hatching, under controlled conditions. Certain cohorts were regularly euthanized, after which specimens were kept intact in 70% ethanol at the Museum National d’Histoire Naturelle, Paris, France. From this wet collection (MNHN.ZMO 2014), we used five specimens for this study: MNHN.ZMO 2014 – 215 and MNHN.ZMO 2014–264 were euthanized at 4 dph; MNHN.ZMO 2014 – 227 at 8 dph; MNHN.ZMO 2014 – 208 at 30 dph; and MNHN.ZMO 2014 – 236 at 50 dph, representing three ontogenetic stages: early, mid‐way and close to fledging. These specimens were raised under the same temperature and light conditions with an *ad libitum* food source (Khabazi, 2017). Except for MNHN.ZMO 2014 – 264, they belonged to the control group of the original study (Khabazi, 2017), in which ducklings were kept in a 200 × 600‐cm enclosure without swimming facilities. In contrast, MNHN.ZMO 2014 – 264 was raised in the swimmer experimental group in a 310 × 400‐cm enclosure supplied with a pool for swimming that was accessible to the animals any time. Specimens from the control group were neither limited nor forced to engage in physical activities, whereas the swimmer group was regularly encouraged to swim 10 times for 10 min a day. For further details on the experimental conditions, see Khabazi (2017).

With respect to the current study and its results, we obtained stained histological slides only of the radius of MNHN.ZMO 2014 – 264, which was the first bone that was prepared and in which the initial observation of chondroid bone was made. However, we do not expect that the swimming activity of MNHN.ZMO 2014 – 264 up to 4 days posthatching age (until its euthanasia) evoked unnatural or pathological osteohistological features in the radius because (1) the locomotor use of the altricially developing forelimbs at this ontogenetic stage is very limited compared with hind limbs in ducklings (Dial & Carrier, 2012); (2) in this experimental setup, specimens from the control group were in fact partially deprived of normal physical activity (swimming), hence the swimming group is a better representative of the natural locomotor repertoire of ducklings; (3) even if chondroid bone is most striking in the radius of the single swimmer specimen, MNHN.ZMO 2014 – 264, our subsequent observations on the limb bones of specimens from the other experimental groups (control group in this study and other experimental groups in preparation) demonstrate the widespread occurrence of chondroid bone, and hence its non‐pathological nature.

### Repository of duck specimens and osteohistological sections

The fore‐ and hindlimbs from one side of the Rouen duck specimens (*Anas platyrhynchos domesticus*) were removed for histological sampling of limb bones. The duck specimens are housed at the Museum National d’Histoire Naturelle, Paris (MNHN), as part of the wet specimen collection obtained and supervised by Dr Anick Abourachid. The removed limbs were processed into osteohistological sections housed at the Department of Biology, Ghent University.

### Dissection and sampling of limb bones

After detaching the fore‐ and hindlimbs at the glenoid and acetabulum, respectively, from one side of each specimen, the humerus, radius, and ulna of the forelimbs, and the femur, tibiotarsus, and tarsometatarsus of the hindlimbs were separated at their joints and defleshed. Bone length was measured with intact articular cartilage. Thereafter, bone shafts were cut in half transversely at mid‐diaphysis with a scalpel in the smallest, least mineralized bones, and with a Dremel rotating saw (0.5‐mm‐thick diamond blade) in larger bones. Except for the smallest forelimb elements at 4 and 8 days, both epi‐metaphyses of the limb bones were cut off to allow dehydrating and defatting solutions and fixatives to penetrate the diaphyseal tissue better.

To acquire a wider range of information of the constituting bone tissues, the proximal halves of the mid‐diaphyseal bone cylinders were prepared as petrographic thin sections and the distal halves as demineralized, microtome‐cut, and stained paraffin sections. In this study, the primary osteohistological tissue identification was based entirely on the demineralized, paraffin‐embedded, and stained sections. Undemineralized petrographic sections were used, on one hand, for the diaphyseal microanatomical measurements to avoid differential tissue expansion artefacts accompanying demineralization, and on the other hand, for comparing the appearance of corresponding tissues identified in demineralized‐stained sections (Supporting Information Fig. S1, Table S1). As fossil bones generally preserve phenotype via heavy diagenetic mineralization processes, osteohistological studies predominantly use undemineralized petrographic sections to identify tissues in extinct vertebrates. Thus, for future comparative evolutionary studies, cross‐checking the visual appearance of the same tissues prepared with these two techniques is a very important initial step to ensure the most reliable tissue identification in fossils.

### Preparation protocol of osteohistological slides

All bone cylinders were fixed, dehydrated, and defatted in the following series of steps: they were put in ethanol 50% and 30% for 10 h each, in phosphate‐buffered saline (PBS) for 2 × 5 h, in formaldehyde 4% for 12.5 days, in PBS for 12 h, in a soap solution (‘Zout’) for 1 day at 50 °C on a shaker, and then in PBS for 1 day.

Proximal bone cylinder halves prepared for undemineralized petrographic sections were dehydrated in a 30–50–70–100% ethanol series for 10–12 h each (2 × 5 to 2 × 6 h in 100%), defatted in xylene for 2 × 5 to 2 × 6 h and replaced into ethanol 100% for 2 × 6 h. They were air dried and embedded in Araldite 2020 epoxy resin. Resin blocks with the exposed mid‐diaphyseal bone cross‐section were mounted on glass slides and cut with a high precision saw (Isomet 1000, Buehler) to ~350‐µm thickness. Sections were manually ground down to 80–60 µm final thickness on a series of SiC powder of 240–400–600–800 grit sizes, and were covered with a glass cover slip.

Distal bone cylinder halves prepared for paraffin‐embedded sections were demineralized in ethylenediaminetetraacetic acid (EDTA) 14% for 3–4 weeks with the solution replaced once. The samples were put back into PBS for 10–12 h, and then into the same ethanol–xylene series as the proximal bone samples. Finally, bone cylinders were put in melted paraffin for 5 h in the first bath and then kept in the melted paraffin for more than 2 weeks until final embedding. The paraffin blocks were sectioned on a rotation microtome (MICROM HM360) with disposable knives (SEC 35p low‐profile blades) to 5‐µm‐thick sections. Sections were deparaffinized in xylene at room temperature for 1 h in the first bath and 2 min in the second bath, and were stained with various techniques (see staining protocols below).

### Staining protocols

#### Toluidine blue (TB)

TB solution was prepared by dissolving 2 g sodium tetraborate in 100 mL distilled water (D H_2_O), after which another 100 mL D H_2_O and 1 g TB powder were added to the solution (pH 9). After filtering this solution, sections were kept in it for 30 s, then rinsed with running tap water for 3 min, dabbed, and dried on a heating plate. Cover slips were adhered by mounting medium and the sections dried at room temperature.

#### Masson’s trichrome (MT)

Sections were immersed in 96% and 70% ethanol, 2 × 2 min each, in D H_2_O for 3 × 2 min, in Mayer’s haematoxylin nuclear stain for 10 min, rinsed with running tap water for 10 min, then kept in Xylidine Ponceau red counterstain for 2 min, in D H_2_O for 3 × 1 min, in 1% phosphomolybdic acid for 5 min, again in D H_2_O for 2 × 1 min, in Light Green SF for 2 min, in D H_2_O for 2 × 30 s, and in ethanol 80% for 10 s and 96% for 2 × 3 s. Finally, sections were immersed in xylene for at least 2 min and covered with DPX mounting medium.

#### Alcian blue/haematoxylin (ABH)

For combined Alcian blue/haematoxylin staining, sections were placed in 3% acetic acid for 3 min, in 1% Alcian blue–3% acetic acid solution for 1 h, rinsed in 3% acetic acid solution and then for 10 min in D H_2_O, stained with Mayer’s haematoxylin for 10 min, flushed in running tap water for 10 min, and then mounted.

#### Picro‐Sirius red (PSR)

The same procedure as for Masson’s Trichrome (and Improved Trichrome) staining was used up to rinsing the haematoxylin‐stained sections with running tap water for 10 min. Thereafter, the sections were immersed in Picro‐Sirius Red stain (0.5 g Sirius red + 500 mL saturated picric acid solution) for 1 min, in 0.5% acetic acid for 3 × 1 min, in 80% ethanol for 10 s, and then dipped quickly in 96% two times. Finally, sections were immersed in xylene for at least 2 min and covered with DPX mounting medium.

### Microscopic analysis

Mid‐diaphyseal osteohistology of the sections was examined under polarized light microscope (Olympus CX22), photographed with a digital microscope camera (Olympus Microscope U‐TVO.5XC), and the images processed in analysis 5.0 software. All subsequent histomorphometric measurements were acquired with imagej 1.48v and the figures assembled in inkscape 0.92.1 free software.

### Quantification of microanatomical parameters

Basic microanatomical parameters (Table S1) were measured in the petrographic section images of sampled bones using the freehand and polygon selection options, and area measurements in imagej (see Fig. S1).

## Results

**Figure 2:**
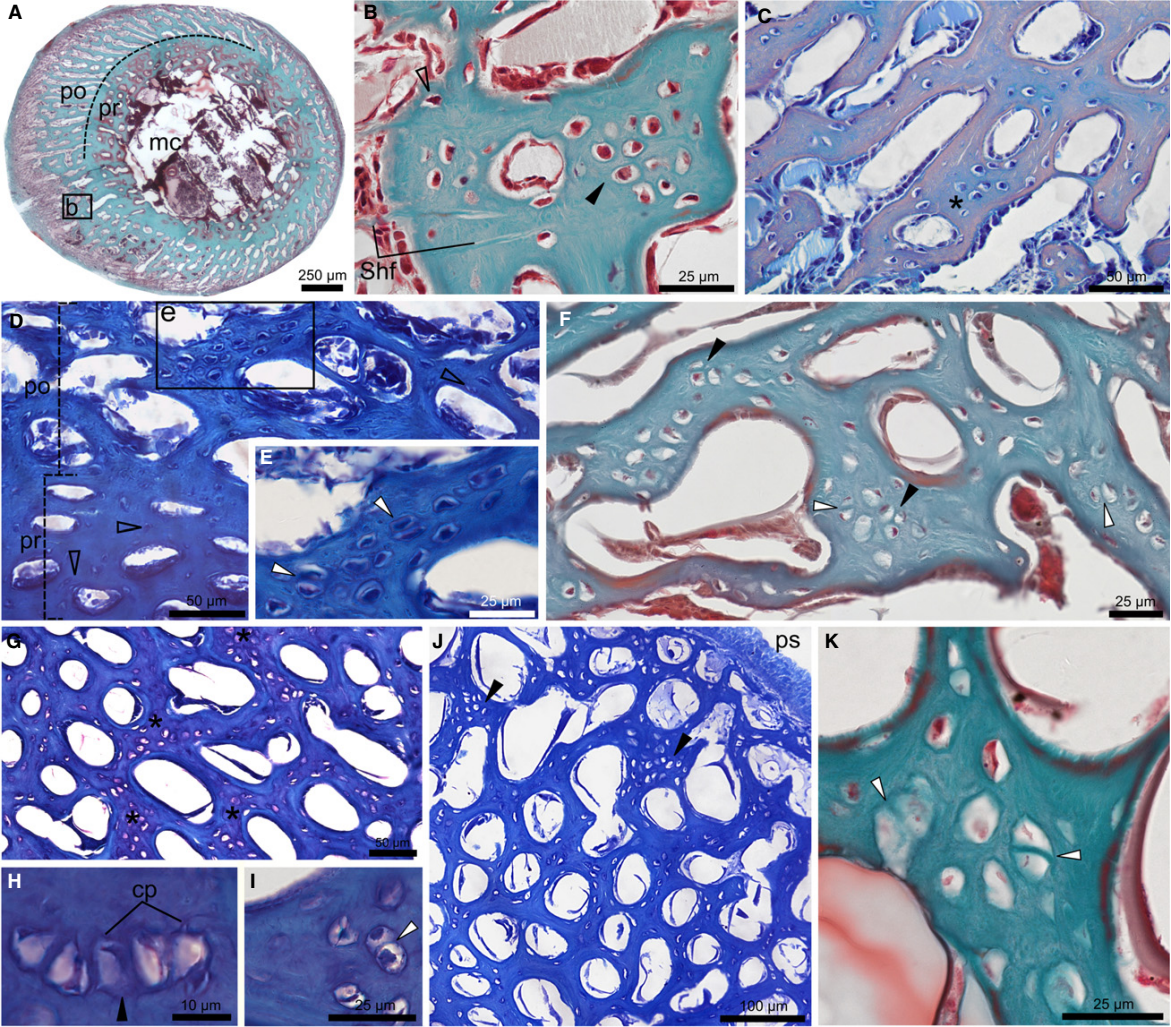
Chondroid bone in the diaphysis of duck limbs at 4 and 8 dph. (A) Femur at 4 dph with MT staining reveals distinct prehatching (pr) and posthatching (po) cortex and medullary cavity (mc). Inset ‘b’ indicates magnified area shown in (B). (B) Chondrocyte‐like cells (black arrowhead) and cells with regular osteocyte morphology (empty arrowhead). Sharpey’s fibres (Shf) invade the cortex. (C) Femur at 4 dph stained with ABH. Moderate blue metachromasia around chondroid cells (asterisk) contrasts peach‐coloured osteonal bone around cavities. (D) Tarsometatarsus at 4 dph with TB staining. Note small osteocytes (empty arrowheads) in ‘pr’ and chondroid cells in ‘po’. Inset ‘e’ indicates magnified area shown in (E). (E) Chondroid bone with enlarged, angular, chondrocyte‐like cells. Pairs of cells apparently share a common lacuna (white arrowheads). (F) Humerus at 8 dph stained with MT shows pairs (white arrowheads) and groups (black arrowheads) of chondroid cells. (G–I) Femur at 8 dph with TB staining with moderate purple metachromasia in chondroid bone areas (asterisk) in (G) and pairs (white arrowhead) and stacks (black arrowhead) of chondroid cells in (H,I). Short cellular processes (cp) protrude from the rounded lacunae (H). (J) Tarsometatarsus at 8 dph stained with TB shows a cortical area where chondroid bone (black arrowheads) is more abundant towards the periosteal surface (ps). (K) Tarsometatarsus at 8 dph stained with MT. Darker green colour around pairs of triangular cells hints at common territorial matrix (white arrowhead)

**Figure 3:**
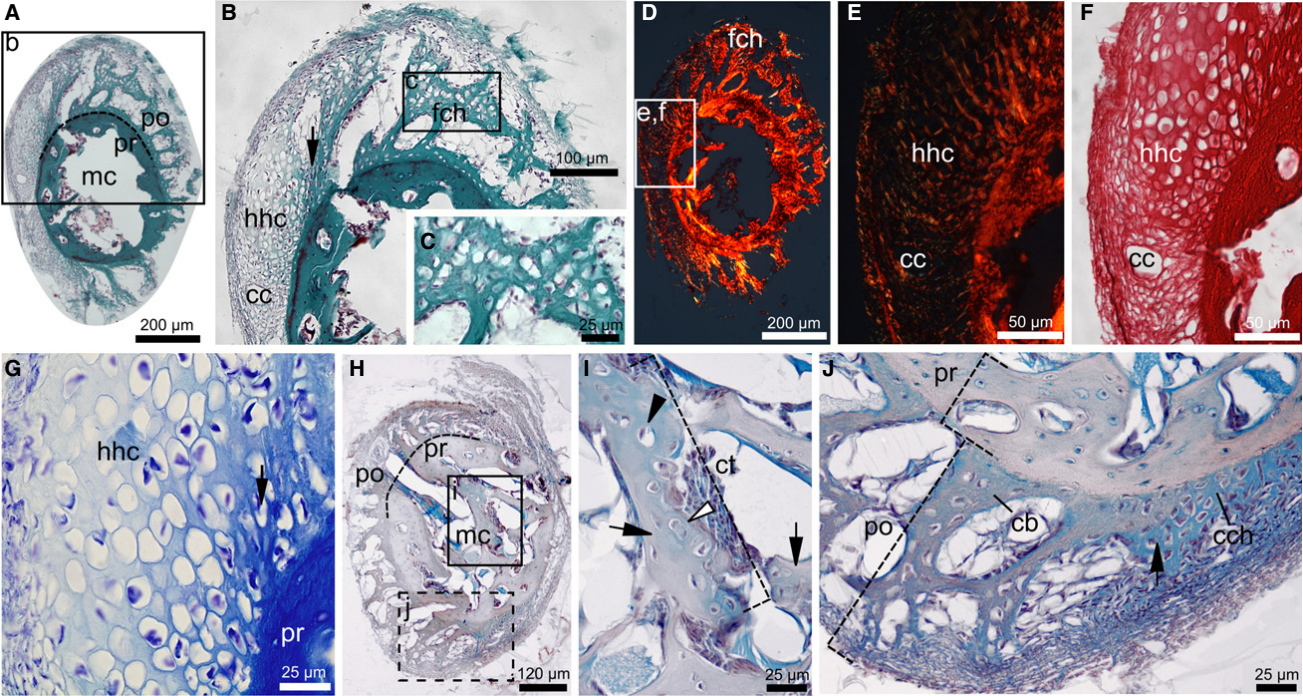
Transient chondroid tissues in the diaphysis of duck radii at 4 dph. (A–G) Radius of MNHN.ZMO 2014–264 with hypertrophied hyaline cartilage (hhc) transitioning into a fibrous chondroid tissue (fch) in the posthatching cortex (po). (A–C) Transient cellular morphologies with MT staining. Insets ‘b’ and ‘c’ in (A) and (B) indicate magnified areas shown in (B) and (C), respectively. (D–F) PSR staining under cross‐polarized light in (D) and (E), and linearly polarized light in (F). Insets ‘e’ and ‘f’ in (D) indicate magnified areas shown in (E) and (F). Birefringence is only absent in ‘hhc’ matrix. Longitudinal cartilage canals (cc) invade ‘hhc’. (G) Closely spaced, swollen cells with very little, opaque matrix in ‘hhc’ with TB staining. Black arrows in (B) and (G) show ‘hhc’ – ‘fch’ transition zone. (H–J) Radius of MNHN.ZMO 2014 – 215 stained with ABH. Insets ‘i’ and ‘j’ in (H) indicate magnified areas shown in (I) and (J). Section in (J) is taken ≤ 10 µm away from (H). (I) Remaining cartilage trabecula (ct) in the forming medullary cavity (mc) contains single (black arrowhead) and paired (white arrowhead) enlarged chondrocytes within the homogeneous blue cartilage matrix. (J) Transition (black arrow) between cell‐rich chondroid tissue (cch) and chondroid bone (cb) where matrix staining also changes from more cartilage‐like (blue, as ‘ct’ in (H)) into more bone‐like (blue with peach shading). Apart from its fibrous ECM, ‘cb’ reveals similar cellular and staining characteristics to ‘ct’, contrasting the bony prehatching cortex (pr)

**Figure 4:**
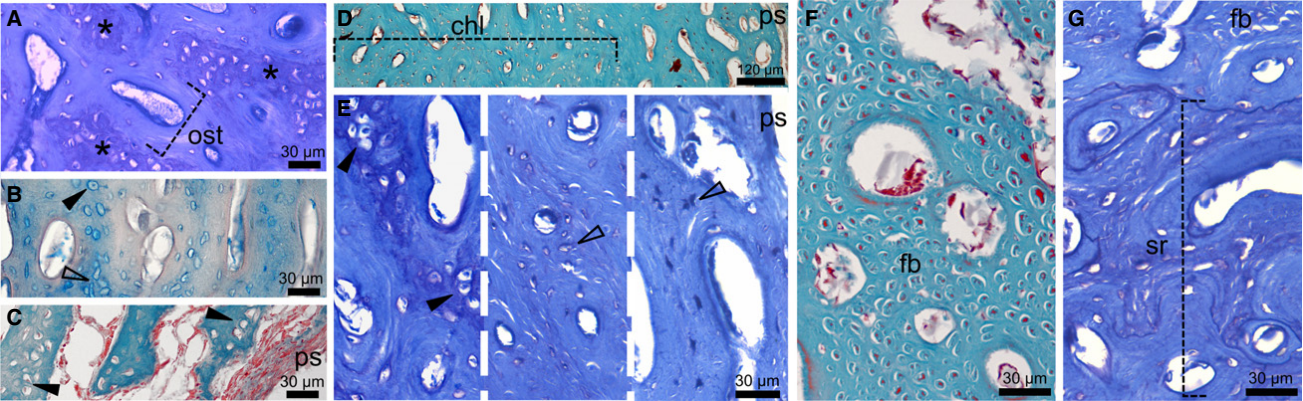
Chondroid bone in the diaphysis of duck limbs at 30 dph. (A) Humerus stained with TB shows that ‘cb’ areas with transitional chondroid cells and slight metachromatic ECM (asterisk) are distinct from osteonal units (ost). (B) Radius with ABH staining shows interosteonal areas with chondroid cells (black arrowhead) and irregularly shaped, woven bone osteocytes (empty arrowhead). (C) Ulna stained with MT exhibits chondroid bone up to the periosteal surface (ps). (D) Chondroid bone‐rich layer (chl) in the inner cortex of the femur stained with MT. (E) Compositional change across the TB‐stained femoral diaphysis showing abundant chondroid cells (black arrowhead) in the inner cortex grading into woven bone osteocytes (empty arrowheads) towards the outer cortex. (F,G) Tarsometatarsus with MT (F) and TB (G) staining. (F) Longitudinally running, cross‐cut fiber bundles (fb) and interspersed cells may originate from fibrocartilage integration. (G) Extensive secondary remodelling (sr) in the inner cortex.

Diaphyseal transverse sections of fore‐ and hindlimb bones of ducklings at 4, 8, and 30 dph revealed interosteonal areas in the periosteal trabeculae of the posthatching cortex that show cartilage‐like characteristics (Figs 2, 3, 4). Morphological diversity of cells and/or lacunae in these areas covers the range from typical hyaline chondrocytes to cells transitioning into irregularly shaped and spaced osteocytes characteristic of static osteogenesis‐derived (SO) woven bone (Marotti et al. 1999; Ferretti et al. 2002; Palumbo et al. 2004; Marotti, 2010). Chondrocyte‐like cells are rounded or bluntly angular with a smooth outline apparently lacking connecting cellular processes (Fig. 2B–E), whereas more transitional cells show a few short cellular processes protruding from the rounded edge of the lacunae, like incipient forms of dendritic osteocyte processes (Fig. 2H). They most frequently lie in pairs or groups of multiple cells in a globular pattern or in a short column‐like stack (Fig. 2E,F,H,I,K), as seen in the isogenous cell groups of hyaline cartilage. Sometimes pairs of cells also seem to share common lacunae (Fig. 2E,I,K), and Masson’s trichrome staining often delineates common territorial matrix around groups of chondroid cells (Fig. 2F,K). Moderate metachromasia of the cell territories and larger areas of the extracellular matrix (ECM) with TB as well as ABH stains indicate the presence of acidic constituents, such as glycosaminoglycans (GAG), further emphasizing similarities with cartilage (Figs 2C,G, 3I, J, and 4A,B). Even though initial fixation methods and storage circumstances of the duck specimens were inadequate for proper analysis of specific chondrocyte and osteoblast markers to further characterize this transitional tissue, based on the aforementioned characteristics, we identify it as chondroid bone.

Concerning its amount and spatial distribution among different elements throughout ontogeny, chondroid bone at early ontogenetic stages (4 and 8 dph) is most abundant in the femur (Fig. 2A–C,G–I), but is also present in considerable amount in the tarsometatarsus (Fig. 2D,E,J,K). In contrast, the well‐developed posthatching cortex of the tibiotarsus, just like the underdeveloped cortex of the ulna, lacks typical chondroid cellular morphology at 4 and 8 dph. The humerus has only low chondroid content at 4 dph and is already much higher at 8 dph (Fig. 2F). One of the two studied radii at 4 dph (MNHN.ZMO 2014 – 264; Fig. 3A–G) reveals a large periosteal area of tissue transition from swollen, hypertrophied chondrocytes with very small, homogeneous‐looking ECM that does not show birefringence (Fig. 3E–G), into an intermediate tissue with irregular chondrocyte‐ and SO‐osteocyte‐like cells and a considerable amount of highly birefringent, fibrous ECM (Fig. 3C,D). This area appears as a transition between the hyaline‐cell cartilage and cell‐rich chondroid bone I categories established for teleost fish intermediate tissues (Witten et al. 2010), albeit with apparently higher ECM fiber content. Although lacking the definite hypertrophied chondrocyte morphology, the homologous cell rich area in the other radius (MNHN.ZMO 2014 – 215; Fig. 3H–J) still contains enlarged chondroid cells with cartilage‐like matrix staining, and it transitions into the typical chondroid bone seen in the other elements which stains intermediate between bone and cartilage (Fig. 3J). In this element, the presence of remnants of resorbed mineralized cartilage trabeculae in the forming medullary cavity (Fig. 3I) further highlights the resemblance in cellular morphology and matrix staining between chondroid bone and cartilage.

At 30 dph (Fig. 4), the periosteal cortex becomes highly fibrous, partially due to the integration of extrinsic (probably metaplastic) fiber bundles within the interosteonal ECM in all studied elements. Groups of chondroid cells appear in the fibrous ECM only in forelimb bones and the femur (Fig. 4A–E). Yet, at this ontogenetic stage chondroid bone is dominated by cells of transitional rather than chondrocyte morphology. The tibiotarsus and tarsometatarsus seem to lack chondroid bone and the areas occupied by cells interspersed among the thick fiber bundles may rather represent integrated mineralized fibrocartilage originating from surrounding connective tissues (Fig. 4F,G). The bulk of chondroid bone appears in the inner half of the thickest cortical regions (Fig. 4D,E), although it reaches the periosteal surface in the ulna (Fig. 4C). Remodelling in the inner cortex of the tarsometatarsus at 30 dph (Fig. 3G) may account for the absence of chondroid bone in this element. By 50 dph, none of the studied elements shows chondroid bone (Fig. S2).

## Discussion

Being an intermediate tissue family representing a continuum of transitions between cartilage and connective tissues, chondroid tissues are hard to define, and hence to identify, on a morphological and/or histochemical basis. This results in a confusing variety of definitions and terminologies. Categories such as chondroid I, II and III (Beresford, 1981), chondroid vs. chondroid bone (Hall & Witten, 2007; Hall, 2014), cartilage Category 3–5 (Witten et al. 2010), and temporary vs. permanent character (Beresford, 1981; Hall & Witten, 2007) aim to capture this diversity. However, these categories overlap with a large number of possible combinations of bony and cartilaginous characters. The many observations and reports on chondroid tissues in various vertebrates date back to the 19th century and cover a range of non‐pathological as well as pathological occurrences. These include transient or permanent tissues in early endochondral and intramembranous skeletogenesis, regions adjacent to articulations, symphyses, tendinous and ligamentous attachment sites, protuberances, developing deer antler, penile bone (baculum), kype in the male salmon, avian medullary bone, healing fractures, ectopic/extraskeletal mineralization, and tumors (for reviews see Beresford, 1981; Hall & Witten, 2007). In our sample, it consistent presence in at least four different individuals of growing ducklings, and its abundance and distribution trends in the limb bones throughout ontogeny exclude a pathological origin for this periosteal chondroid tissue.

Nevertheless, apart from MNHN.ZMO 2014 – 264, the lack of swimming facilities in the original enclosure of our studied specimens raises the question of how this deficiency in the natural locomotor repertoire of ducks might have affected their limb bone tissues. It is well known that different types and magnitudes of loading will affect growing bone in many different ways (e.g. Biewener et al. 1989; Biewener & Bertram, 1993; Robling et al. 2001, 2006; Starck & Chinsamy, 2001; Lieberman et al. 2003; Iwamoto et al. 2004; Montes et al. 2005; Vicente‐Rodríguez, 2006; Foutz et al. 2007), and we do not yet know how swimming deprivation influences limb bone histology in growing mallards. For instance, it is expected that ducks forced to stay on the ground permanently carrying their bodyweight will experience more extensive loading (in duration as well as in magnitude) on their hindlimbs than will ducks that can spend a substantial amount of time in the water, where no body mass‐related loads affect their limbs. However, even if hindlimbs were loaded above natural levels for amphibious ducks due to this forced terrestriality (although still well within their physiological regimens), forelimb elements, which do not essentially participate in terrestrial locomotion and hence should not be affected by this, also possess chondroid bone. Thus, it can be safely assumed that swimming deprivation has little to no effect on the presence of chondroid bone in our duck samples.

In the limb bone shafts of a number of tetrapods, including small mammals, birds and even a frog, a layer of chondroid bone I (*sensu* Beresford, 1981) that is only a few cells thin, has been reported to be deposited as a transitional tissue between the cartilage precursor and the periosteal bone in the early phase of diaphyseal ossification (Beresford, 1981). This layer quickly disappears by resorption and remodelling during growth. Although regarded as largely inconsistent, some early reports on subperiosteal chondrogenesis in the growing limb bone shaft of small mammals exist (see review of Zawisch‐Ossenitz’s work in Beresford, 1981). However, extensive involvement of periosteal chondroid bone in normal post‐embryonic diaphyseal bone formation with an ontogenetically extended deposition period (at least up to 30 dph, about halfway to adult size), as shown in this study, has not been documented before. Furthermore, as diametric growth progresses in the duckling limb bones, areas of higher chondroid bone content are not preferentially remodelled, unlike mineralized cartilage in endochondral ossification (Ballock & O’Keefe, 2003). Instead, chondroid bone remains the functional part of the juvenile primary limb bone cortex until medullary cavity expansion and/or mechanical demands mediate its resorption along with the rest of primary cortical tissues.

The stimulus initiating the deposition of chondroid tissues (oxygen tension, biomechanical stimulus; Pauwels, 1960; Dhem et al. 1989; O’Driscoll et al. 1997) and the way they originate (direct deposition by a ‘perichondroosteum’, direct metaplasia, mineralization of cartilaginous metaplastic tissues; Beresford, 1981) might be as diverse as their morphological and histochemical composition. However, in all non‐pathological cases, the functional significance of chondroid bone formation lies either in its transitional nature to establish physical connection between bone and cartilage or in its capacity for fast skeletal growth (Huysseune & Verraes, 1986; Huysseune & Sire, 1990; Hall, 2014). Cartilage can expand by interstitial as well as appositional growth, which may be the key in its role of rapid skeletogenesis (Starck, 1994; Montes et al. 2005). The presence of apparently isogenous cell groups in the chondroid bone of duckling limbs implies that cell division, and hence cartilage‐like interstitial growth, took place within a not‐yet mineralized ECM of the forming periosteal trabeculae. This would combine fast initial growth of cartilage and skeletal stiffness of bone by subsequent mineralization. The chondrogenic potential of the periosteum and chondrocyte transdifferentiation to osteoblasts are well‐documented in skeletal repair and *in vitro* (O’Driscoll et al. 2001; Yoshimura et al. 2006; Hu et al. 2017), but also under normal conditions (Nah et al. 2000; Giovannone et al. 2019). Hence, our case most likely represents an extended transient chondrogenic phase in the periosteal intramembranous ossification of the limb bone shafts resulting in substantial chondroid bone content. If interstitial as well as appositional growth occurs in chondroid bone, they can complement the *in situ* transformation and embedding of SO‐derived osteoblasts (Ferretti et al. 2002; Palumbo et al. 2004; Marotti, 2010) in the intramembranous ossification centers of the well‐vascularized bone collar mesenchyme. This extra growth capacity of chondroid bone could facilitate diaphyseal cortex expansion even further than expected from pure SO‐derived woven bone formation. Thus, the chondroid bone–woven bone composition most likely results in the extremely fast diametric limb bone growth generally seen in birds, among which we find the fastest growing extant tetrapods (Case, 1978).

The distribution and abundance of chondroid bone among different elements through duckling ontogeny largely parallel the respective growth burst periods of the altricial wing and precocial leg bones (Carrier & Leon, 1990; Castanet et al. 1996; Montes et al. 2005; Dial & Carrier, 2012) (Fig. S2, Table S1). This supports its functional interpretation in growth acceleration. Intriguingly, however, the tibiotarsus seems to lack definite chondroid bone in any age category, even though it is the largest limb element and its posthatching periosteal cortex formation is very extensive (both in relative and absolute measures) in early ontogeny (Fig. S1E,F, Table S1). Whether and how the special loading regimens of different avian hindlimb elements (Cubo et al. 1999; Cubo & Casinos, 2000; Casinos & Cubo, 2001; Main & Biewener, 2007; Brassey et al. 2013; Marelli & Simons, 2014) and the complex trade‐offs between precocial function and growth (Carrier & Leon, 1990; Castanet et al. 1996; Montes et al. 2005; Dial & Carrier, 2012) relate to the prominent differences in chondroid bone content between the femur and tibiotarsus is unclear. As the tibiotarsus was shown to have the highest bending strength and Young’s modulus among hindlimb elements in birds (Casinos & Cubo, 2001), it may well be that bodyweight‐related loads, which could have been further increased by forced terrestriality, influenced the relative amount, and hence the lack of definite chondroid areas in the tibiotarsus. Although its abundance in the femur still allows hindlimb precocity, the mechanical properties of chondroid bone need to be studied to assess its possible effect on bone strength and skeletal stiffness in the mechanically challenging environment of precocial locomotion.

Finally, because chondroid bone in the growing duck limbs most likely promotes fast diametric bone growth, it is expected to occur generally in birds and potentially in other extant and extinct fast‐growing tetrapods, like some non‐avian dinosaurs, other archosaurs and therapsids (mammals and their ancestors). Although lacunar morphology in fossil bones strongly depends on preservation state, calcified cartilage, metaplastic tissues, and even chondroid bone have already been identified in fossils (Horner et al. 2000, 2016; Horner & Goodwin, 2009; Johanson et al. 2010; Bailleul et al. 2013, 2016). This suggests that careful examination could reveal the presence of chondroid bone in extinct vertebrates, too. If so, chondroid bone may provide further clues to how extremely large body sizes seen in many fossil taxa could have been achieved by very fast growth. Future studies are thus encouraged to appropriately explore the nature and phylogenetic distribution of chondroid bone in the periosteal cortex of developing limbs and its relationship with the precocial–altricial strategies. First, diverse species of birds following different strategies along the precocial–altricial developmental spectrum will have to be checked for the presence, amount, and distribution pattern of chondroid bone in the limb bone shafts throughout ontogeny. Considering its suggested role in fast bone growth, chondroid bone should also be sought in the limb bones of other modern fast‐growing tetrapods (e.g. mammals) to infer its extant phylogenetic distribution pattern. For this, more in‐depth analysis of tissue characteristics revealing extracellular matrix composition and chondroid cell divisions in early stages of chondroid bone formation (e.g. by using immunohistochemistry, flow cytometry and other techniques for matrix and cell cycle analysis) is needed to verify tissue identity and its potential for initial interstitial growth. Other specific techniques (e.g. gene expression studies, cell lineage tracing) are also necessary to explore the possibility of a potential *in situ* transdifferentiation of chondroid cells into woven bone osteoblasts – osteocytes. Finally, with this firm extant comparative basis, it becomes possible to explore the presence, amount, and distribution pattern of chondroid bone in extinct, fast‐growing vertebrates which will provide a proper input dataset for a well‐informed phylogenetic analysis. This step also requires a deeper comparative morphological (and potentially histochemical) analysis of ground sections of extant and fossil bones to allow a confident identification of chondroid bone in fossil tissues as well.

Such a holistic approach will provide substantial insight into the evolutionary origin and functional significance of the extensive integration of chondroid bone into the appendicular diaphyseal skeletogenesis in tetrapods.

## Conflict of interest

The authors have no conflict of interest to declare.

## Author contributions

E.P. conceived and designed the study and methodology, dissected the ducks, prepared the bone samples, investigated, photographed, measured and evaluated the sections, prepared the figures and wrote the manuscript. P.E.W. participated in conceptualizing the methodology, section staining and evaluating and taking pictures of the sections, checked and edited final draft. A.A. acquired and currently curates the duck specimens used in this study, checked and edited final draft. A.H. helped conceptualize the section staining methodology and evaluate the sections, checked and edited final draft. D.A. provided necessary lab facilities, checked and edited final draft. All authors gave final approval for publication and agree to be held accountable for the work performed therein.

## Supporting information


**Fig. S1.** Utilization of undemineralized petrographic diaphyseal transverse sections in this study.

**Fig. S2.** Diaphyseal osteohistology of duck limb bones at 50 dph.

**Table S1.** Microanatomical parameters measured and calculated on undemineralized petrographic diaphyseal transverse sections of duck limb bones.

## Data Availability

The data that support the findings of this study are partly available at the histological section repository of the Department of Biology, Ghent University, and from the corresponding author upon reasonable request.
