# Supplementary figures and images for: Extensive chondroid bone in juvenile duck limbs hints at accelerated growth mechanism in avian skeletogenesis
Source: J Anat. 2019 Oct 31;236(3):463–73. doi: 10.1111/joa.13109 (PMC7018642; doi:10.1111/joa.13109)

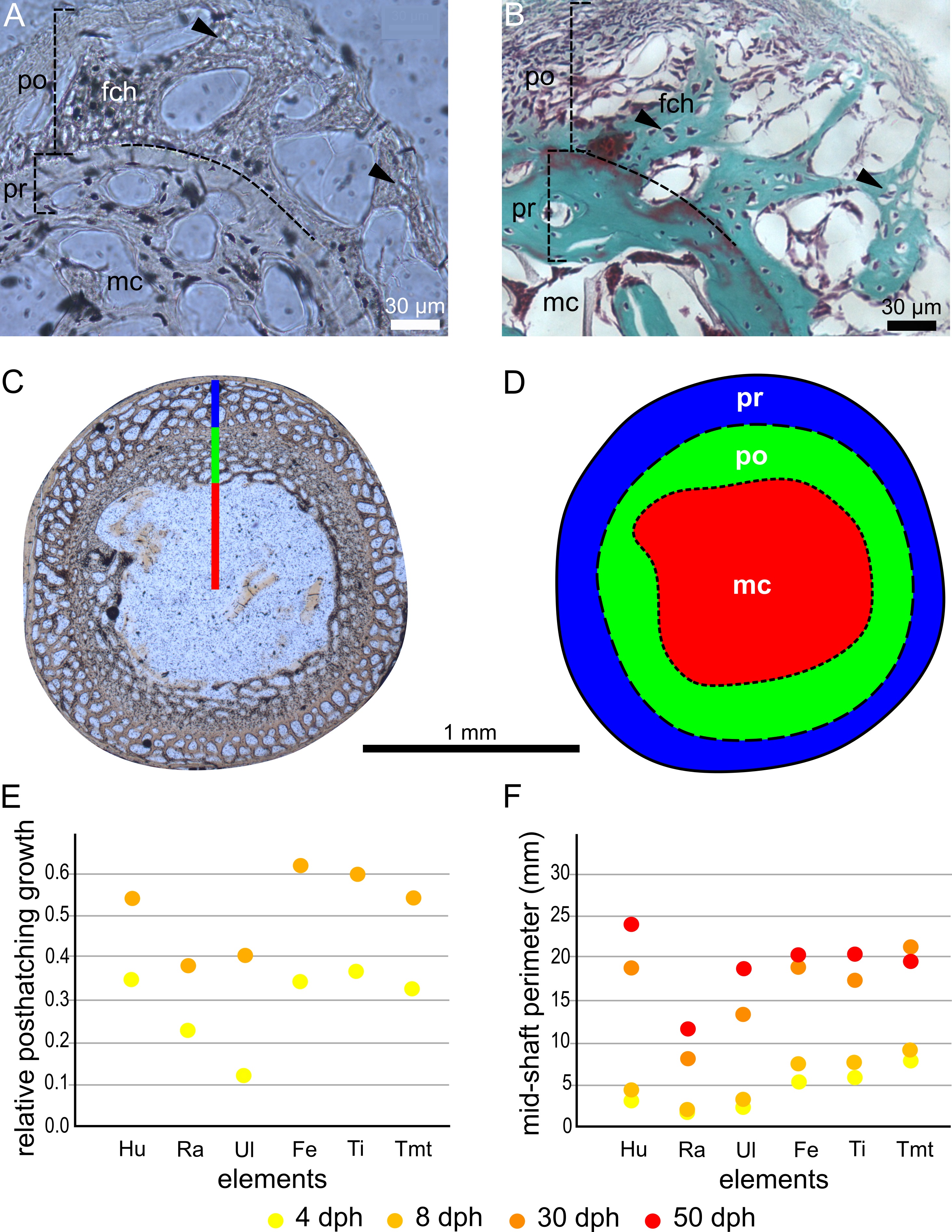

Supplement: Supplementary file 1 — Fig. S1. Utilization of undemineralized petrographic diaphyseal transverse sections in this study. [file JOA-236-463-s001.jpg]

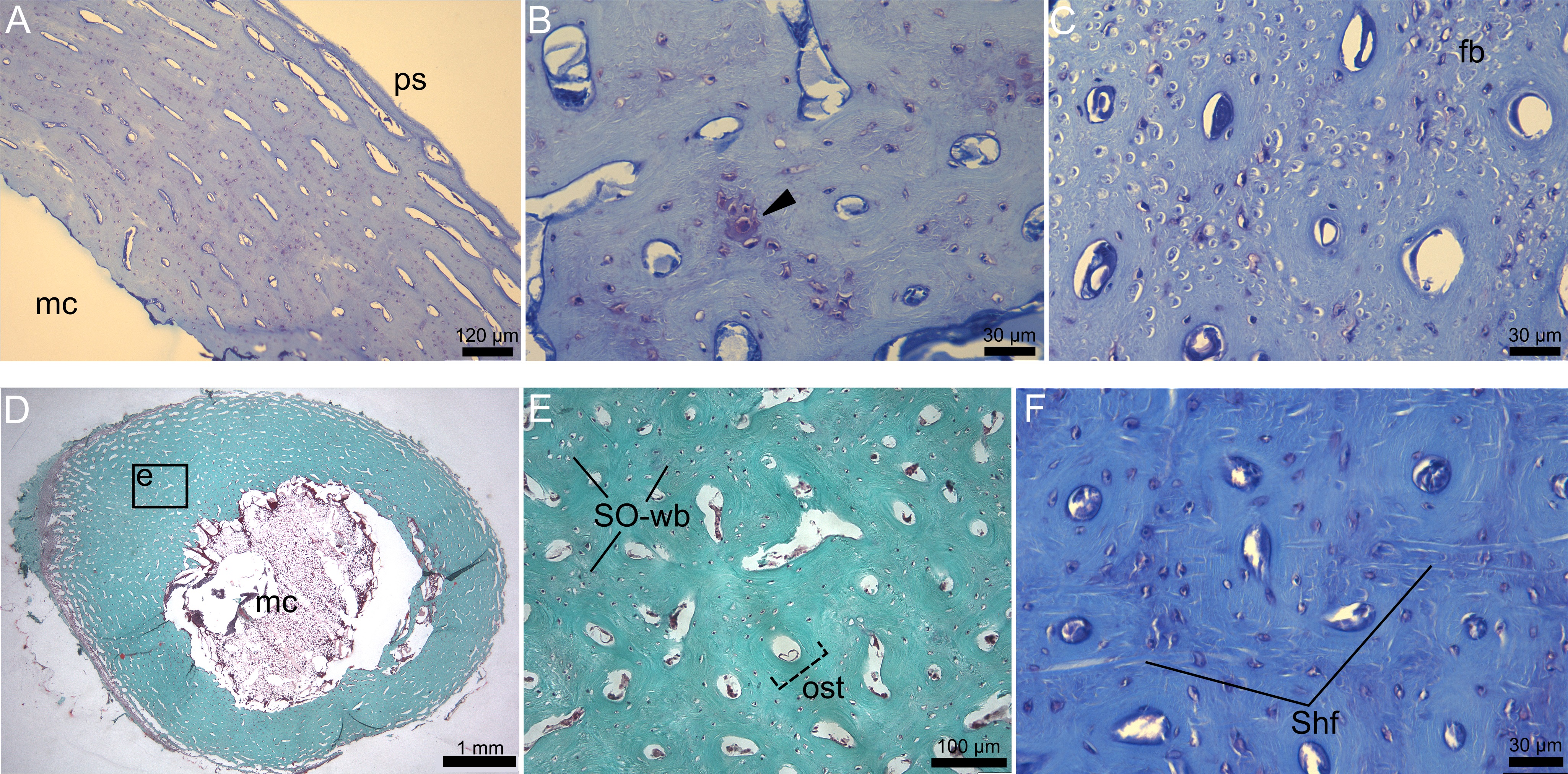

Supplement: Supplementary file 2 — Fig. S2. Diaphyseal osteohistology of duck limb bones at 50 dph. [file JOA-236-463-s002.jpg]
