# Supplementary material for: Extensive chondroid bone in juvenile duck limbs hints at accelerated growth mechanism in avian skeletogenesis
Source: J Anat. 2019 Oct 31;236(3):463–73. doi: 10.1111/joa.13109 (PMC7018642; doi:10.1111/joa.13109)
Supplement: Supplementary file 3 — Table S1. Microanatomical parameters measured and calculated on undemineralized petrographic diaphyseal transverse sections of duck limb bones. [file JOA-236-463-s003.docx]

| **Duck ID** | **Age (days post-hatching)** | **Studied element** | **Cortex perimeter (mm)** | **Total cross section area (mm^2^)** | **Posthatching cortex area (mm2)** | **Relative posthatching growth** |
| --- | --- | --- | --- | --- | --- | --- |
| MNHN.ZMO 2014 – 264 | 4 | Ra | 1.95 | 0.26 | 0.1 | 0.38 |
| MNHN.ZMO 2014 – 215 | 4 | Hu | 3.09 | 0.76 | 0.26 | 0.34 |
|  |  | Ra | 1.79 | 0.24 | 0.05 | 0.23 |
|  |  | Ul | 2.21 | 0.41 | 0.05 | 0.12 |
|  |  | Fe | 5.37 | 2.2 | 0.75 | 0.34 |
|  |  | Ti | 5.66 | 2.51 | 0.92 | 0.37 |
|  |  | Tmt | 7.74 | 4.3 | 1.38 | 0.32 |
| MNHN.ZMO 2014 – 227 | 8 | Hu | 4.29 | 1.5 | 0.81 | 0.54 |
|  |  | Ra | 1.84 | 0.26 | 0.1 | 0.38 |
|  |  | Ul | 2.87 | 0.67 | 0.27 | 0.40 |
|  |  | Fe | 7.2 | 4.02 | 2.49 | 0.62 |
|  |  | Ti | 7.32 | 4.22 | 2.52 | 0.60 |
|  |  | Tmt | 8.89 | 6 | 3.27 | 0.55 |
| MNHN.ZMO 2014 – 208 | 30 | Hu | 18.79 | 26.3 | NA | |
|  |  | Ra | 7.97 | 5.04 |  |  |
|  |  | Ul | 13.16 | 14 |  |  |
|  |  | Fe | 18.7 | 27.1 |  |  |
|  |  | Ti | 17.32 | 23.35 |  |  |
|  |  | Tmt | 21.18 | 34.2 |  |  |
| MNHN.ZMO 2014 – 236 | 50 | Hu | 23.95 | 44.3 | NA | |
|  |  | Ra | 11.69 | 10.29 |  |  |
|  |  | Ul | 18.7 | 27.1 |  |  |
|  |  | Fe | 20.37 | 31.8 |  |  |
|  |  | Ti | 20.47 | 31.4 |  |  |
|  |  | Tmt | 19.59 | 28.58 |  |  |

**Table S1. Microanatomical parameters measured and calculated on undemineralized petrographic diaphyseal transverse sections of duck limb bones.** Relative posthatching diametric growth is calculated as the ratio of posthatching cortex area to total cross section area (including medullary cavity). Light green and blue shading of cells indicate wing and leg elements, respectively. Of MNHN.ZMO 2014 – 264, we only had a radius sample for this study. Abbreviations: Fe, femur; Hu, humerus; Ra, radius; Ti, tibiotarsus; Tmt, tarsometatarsus; Ul, ulna
